# Sir G. Stephen on the Plea of Monomania

**Published:** 1848-07-01

**Authors:** George Stephen


### THE PLEA OF MONOMANIA IN CRIMINAL CASES.

BY SIR GEORGE STEPHEN.

We give the following sketch from Sir George Stephen's "Juryman's Guide," as illustrative of the opinion of an intelligent jurist on the plea of monomania in criminal cases.

"All criminality depends on intention, or on a carelessness which amounts to gross negligence: thus, in the case of shooting a man, the absence of intention to kill will relieve the charge from the guilt of murder; but yet the careless discharge of a loaded pistol, should the evidence reduce the case to carelessness, will amount to manslaughter if the victim dies. To utter a bad shilling in ignorance of its character, to fire a house accidentally, or to administer poison under the impression that it is the right medicine sent by the apothecary, are all guiltless acts, however mischievous or dangerous, because the bad intention is wanting; yet there may be a felonious intention, though not that which the act itself would seem to indicate: thus a man may accidentally kill A in shooting with the intention to murder B, and in such a case, though A was accidentally killed, it still is murder. And if a man breaks into a house with the purpose of robbing it, and being resisted kills the inhabitant, it is murder, although he may have killed him in self-defence. This substitution of intention is based on the principle that every man is reasonably liable for the natural consequences of a felonious purpose carried into execution.

"This is an important principle to understand, for it leads to important practical consequences; first, that evidence of an intention ought always to be given; but secondly, that if the act itself is clearly established, a malicious intention must sometimes be inferred, though not actually proved in the form peculiar to the crime.

"The first of these positions is very valuable to a juryman, to preserve him at once from the painful apprehension of convicting an innocent man, and from the culpable weakness of acquitting one whom he believes guilty, lest, perchance, he may make a mistake; it is to relieve himself from this dilemma that a juryman frequently, in these times, lends a ready ear to the very suspicious defence of insanity; it affords a middle course, with which conscience is easily satisfied; that insanity which can excuse a felonious act must amount to incapacity to distinguish between right and wrong; and if this discriminating power is retained in respect of every act or feeling but one, the fair and reasonable inference is, that on the excepted matter the mind has indulged vindictive or licentious passion so long and so habitually, as to have become callous on that particular point; but this habitual self-indulgence is common to every crime, and is of itself the essence of criminality, though no detected overt act may bring out the intention so as to make it cognizable by law. The law punishes crime, because the act indicates intention; or rather, because it exposes intention, which may be proved by evidence dehors the act itself; the killing, coupled with other evidence of intention, becomes murder; without the evidence of intention, it is chance-medley. The secret indulgence of any passion cannot in common sense be separated from a purpose of gratifying it, when opportunity serves of gratifying it with impunity; by long indulgence the passion gains the mastery over prudence or apprehension; or, in other words, over reason; and then the man yields to it, commits a crime, and is pronounced a monomaniac. Men of science declare that monomania is consistent with sanity in all other points, and a jury acquits; we may be in error, but in our view such monomania amounts only to this, that a long-cherished feeling of malignity, or of criminal desire, has at length burst the bounds of common sense—as all criminality of desire, if not resisted in its incipient stages, invariably does—and having obtained liberty to range, plunges its self-immolated victim into an abyss, as the herd of swine were precipitated into the sea by the legion of devils, when these same devils were once let loose to take their course. Such monomania is not peculiar to the offence of murder, though it is in murder only, because it is now almost the only capital crime, that juries give credence to it. Revenge may lie still for years, and at last will find the opportunity of retaliation; avarice may be concealed till life is far advanced, and then will commit fraud or forgery; sensuality may indulge itself in unobserved secrecy till the head grows grey, and at last will break out in felonious violence: yet in these cases we do not extenuate criminality on the ground of lunacy, unless in other respects the offender betrays irrationality; in some sense every man is irrational who incurs penalty or even disgrace by open self-indulgence; but the mere irrationality of not calculating consequences would excuse the drunkard, the poacher, the pickpocket, and every offender that ever yet was arraigned on a first charge.

"Nor is monomania, if such it must be called, peculiar to crime. There are very few men of active minds who are totally exempt from the habitual indulgence of some whim or fancy, which strengthens as life advances, and the gratification of which, at last, becomes essential to comfort, if not to happiness. Some select benevolent pursuits, as schools, visiting societies, or repositories. Some late distinguished men carried anti-slavery enthusiasm to a monomaniacal pitch; we ourselves confess to a failing that way. We know an excellent man near Sheffield, who has lived on lotteries and chimney-sweepers all his life; even the slave, though equally black, enjoyed but a subordinate portion of his affections. The only essential difference between such cases and the monomania of our criminal courts, is, that legitimate passion may in the one be indulged to the extent of folly, but takes a direction in which it cannot fall into crime; in the other it becomes criminal, because its direction is originally wrong: in both cases it is still passion, and not often abstractedly wrong. The desire of accumulating property, for instance, is at the root of all industry and scientific excellence, and therefore laudable; though when indulged beyond control, it fills our gaols and penitentiaries. Self-vindication is as permissible as self-defence, and, indeed, only another form of it; yet when carried to vindictiveness, it leads to violence and murder. The gratification of animal desires is not only excusable but essential to existence; yet when gratification is sought, regardless of consequences, it leads to brutal force and plunder. Men who habitually practise self-control, not merely over their acts, but over their inclinations, are never betrayed into such excesses; but those who allow thought to ramble at pleasure into excess, give the reins to passion, till it becomes uncontrollable, and sets consequence at defiance; this, in the slang of science, is called monomania; or in simple English, lunacy on a single subject. We are more inclined to view it as the natural result of long-cherished, though long-concealed passion, till it has worked itself up into depravity; and this is the characteristic of all crime: *nemo repente fuit turpissimus.*

"Where this kind of single-eyed insanity, this self-concentration on a criminal object, is such as to deserve the name, and therefore to entitle itself to the impunity of lunacy, there will be found a remarkable peculiarity about it, a peculiarity quite sufficient to distinguish the real from the pseudo affection. The momentary gratification of the desire does not extinguish the existence of it. If murder is the disposition, then one attempt will be followed up by another. If theft is the fancy, then the monomaniac will pilfer in his own house, and from his children, guests, or servants, yet more frequently than in a tradesman's shop. If his taste lies in the incendiary direction, one fire in the course of life will by no means satisfy him. As for example: at the present assizes at Norwich, a man of the name of Frost has been acquitted of murder on the ground of insanity, nor could a clearer case have been established; but he was not content with killing one child, he destroyed all four. His idea seems to have been, that he thus obtained for them an early translation into heaven, and therefore his lunacy was consistent with itself, and he wished to send them all there. But suppose that on the same pretext he had killed only one, and that one the least favourite of them all, and for whose happiness he might therefore be supposed least anxious to provide, would it have been safe in that case to have acquitted him? We think not; unless he had in some way or other indicated that his intention respecting the others was only suspended. We own that we cannot attribute the fuss that has been made about monomania during the last two years to any new lights that have been thrown on the nature or structure of the mind. We are far more inclined to ascribe it to that sickly humanity for which our juries have latterly become proverbial, and which generally has crept more into fashion than quite becomes the sturdy manliness for which our countrymen have long been celebrated. Our fathers and grandfathers troubled their heads but little with such subtleties in criminal proceedings; if their practice was less remarkable for its humanity, it certainly was more distinguished by good sense than our own."

